# 1,1′-(Ethane-1,2-di­yl)bis­(1,4,7-triazonane)

**DOI:** 10.1107/S1600536810019562

**Published:** 2010-05-29

**Authors:** James C. Knight, Ian A. Fallis

**Affiliations:** a Main Building, School of Chemistry, Cardiff University, Park Place, Cardiff CF10 3AT, Wales

## Abstract

In the centrosymmetric title compound (dtne), C_14_H_32_N_6_, two 1,4,7-triaza­cyclo­nonane (tacn, or 1,4,7-triazonane) moieties are linked together each at an amino position by a single ethyl­ene spacer. The mol­ecular packing is supported by pairs of inter­molecular N—H⋯N hydrogen bonds, which form *R*_2_^2^(22) ring motifs and link the mol­ecules into infinite chains running parallel to the *a* axis.

## Related literature

For an investigation into the coordination chemistry of dtne derivatives and similarly bridged polyaza macrocyclic frameworks, see: Schröder *et al.* (2000 ▶). For dinuclear metal complexes of related ligands, see: Sinnecker *et al.* (2004 ▶); Marlin *et al.* (2005 ▶). For the crystal structure of the related compound 1,4,7-triaza­cyclo­nonane (tacn), see: Battle *et al.* (2005 ▶). For the structures of other metal complexes of dtne, see: Li *et al.* (2009 ▶). For hydrogen-bond motifs, see: Bernstein *et al.* (1995 ▶). For the preparation of a similar compound, see: Burdinski *et al.* (2000 ▶).
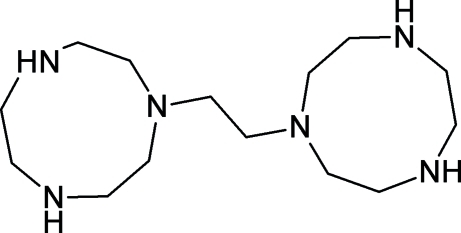


## Experimental

### 

#### Crystal data

C_14_H_32_N_6_
*M* *_r_* = 284.46
Triclinic, 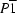

*a* = 6.2732 (3) Å
*b* = 6.4988 (3) Å
*c* = 10.7152 (6) Å
α = 99.751 (2)°
β = 93.115 (2)°
γ = 110.410 (3)°
*V* = 400.45 (3) Å^3^
*Z* = 1
Mo *K*α radiation
μ = 0.08 mm^−1^
*T* = 150 K
0.4 × 0.28 × 0.28 mm

#### Data collection

Bruker–Nonius KappaCCD diffractometer
Absorption correction: multi-scan (*SORTAV*; Blessing, 1995 ▶) *T*_min_ = 0.649, *T*_max_ = 0.985
4952 measured reflections
1806 independent reflections
1599 reflections with *I* > 2σ(*I*)
*R*_int_ = 0.099

#### Refinement

*R*[*F* ^2^ > 2σ(*F* ^2^)] = 0.068
*wR*(*F* ^2^) = 0.208
*S* = 1.23
1806 reflections
99 parameters
H atoms treated by a mixture of independent and constrained refinement
Δρ_max_ = 0.30 e Å^−3^
Δρ_min_ = −0.33 e Å^−3^

### 

Data collection: *COLLECT* (Nonius, 2000 ▶); cell refinement: *SCALEPACK* (Otwinowski & Minor, 1997 ▶); data reduction: *DENZO* (Otwinowski & Minor, 1997 ▶) and *SCALEPACK*; program(s) used to solve structure: *SIR92* (Altomare *et al.*, 1993 ▶); program(s) used to refine structure: *SHELXL97* (Sheldrick, 2008 ▶); molecular graphics: *X-SEED* (Barbour, 2001 ▶); software used to prepare material for publication: *WinGX* (Farrugia, 1999 ▶).

## Supplementary Material

Crystal structure: contains datablocks global, I. DOI: 10.1107/S1600536810019562/sj5006sup1.cif

Structure factors: contains datablocks I. DOI: 10.1107/S1600536810019562/sj5006Isup2.hkl

Additional supplementary materials:  crystallographic information; 3D view; checkCIF report

## Figures and Tables

**Table 1:** Hydrogen-bond geometry (Å, °)

*D*—H⋯*A*	*D*—H	H⋯*A*	*D*⋯*A*	*D*—H⋯*A*
N2—H2⋯N1^i^	0.80 (3)	2.37 (3)	3.129 (3)	159 (2)

Symmetry code: (i) 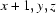.

**Fig. 1.**
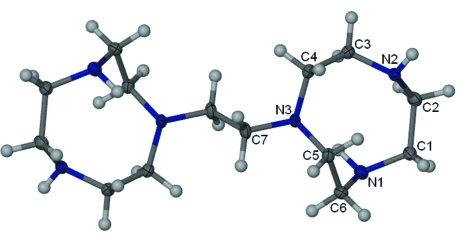
Perspective view of the asymmetric unit, showing the atom numbering. Displacement ellipsoids are at the 50% probablility level. H atoms are represented by circles of arbitrary size. Unlabelled atoms are related to labelled atoms by the symmetry operation -x, -y, -z.

**Fig. 2.**
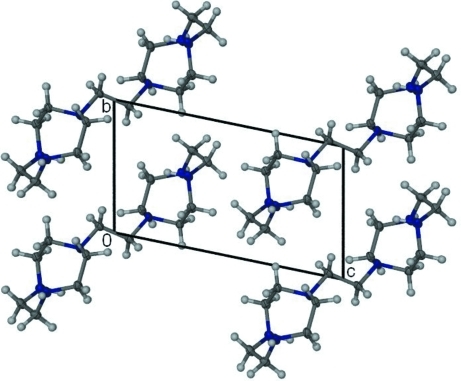
The crystal packing, viewed along the a axis.

**Fig. 3.**
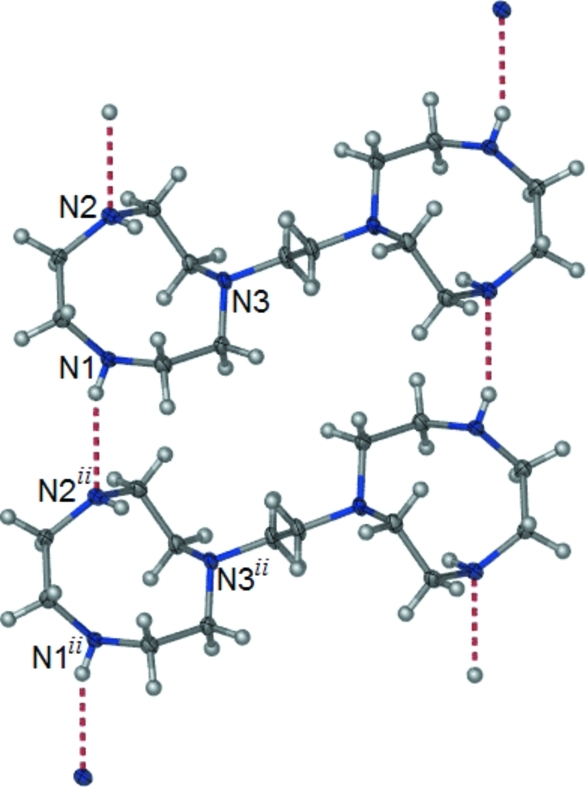
A fragment of the molecular packing, clearly showing H-bond interactions between adjacent molecules. [Symmetry code: (ii) x-1, y, z.].

## supplementary crystallographic information

### Comment

The coordination chemistry of ligand frameworks which contain two tacn moieties linked by two to six carbon atoms has been extensively studied (Schröder *et al.*, 2000). The ability of these so-called "earmuff" ligands to form dinuclear metal complexes, in which two metal centres lie in close proximity, has provided a useful means of investigating the active sites of various biological systems. For example, the dinuclear manganese complexes of ligands dtne (Sinnecker *et al.*, 2004) and 1,2-bis(4,7-dimethyl-1,4,7-triaza-1-cyclononyl)ethane (Me_4_dtne) (Marlin *et al.*, 2005) have received particular attention as a means of investigating Photosystem II. Whilst crystal structures of several dtne transition metal complexes have been reported (Li *et al.*, 2009), the structure of the free ligand in the solid state has, until now, remained elusive.

We can report that dtne crystallizes in the triclinic space group *P -1* with one molecule in the unit cell. The asymmetric unit contains one-half molecule with the other half generated by a centre of inversion which lies at the midpoint of the C7—C7^i^ bond [Symmetry code: (i) = -x, -y, -z] (Figure 1). The bond lengths and angles within each tacn moiety are comparable to those found in the crystal structure of 1,4,7-triazacyclononane hemihydrate (Battle *et al.*, 2005). The N3—C7—C7^i^ bond angle is 112.12 (15) ° which indicates no significant stretching or compression of the ethylene bridge. The molecular packing (Figure 2) is supported by pairs of N—H···N hydrogen bonds between N1 and N2^ii^ [Symmetry code: (ii) = x-1, y, z] (Figure 3). These H-bond interactions generate R_2_^2^(22) ring motifs (Bernstein *et al.*, 1995) and link the molecules into supramolecular one-dimensional chains which run parallel to the a-axis.

### Experimental

1,2-Bis(1,4,7-triaza-1-cyclononyl)ethane, commonly referred to by the abbreviation dtne, was prepared by a modification of the procedure for that of 1,2-bis(4-methyl-1,4,7-triazacyclononyl)ethane (Me4dtne) reported by Burdinski *et al.*, 2000. To a stirred solution of 1,4,7-triazatricyclo[5.2.1.0^4,10^]decane (6.96 g, 5 mmol) in dry acetonitrile (25 ml) was added 1,2-dibromoethane (4.51 g, 2.4 mmol). After 5 days an off-white hygroscopic precipitate was collected by filtration and subsequently dissolved in 6 M hydrochloric acid (100 ml). The resulting solution was heated at reflux for 3 days after which the solvent was removed by evaporation under reduced pressure. The title compound was isolated by the addition of 10 M NaOH (20 ml) and subsequent removal of water by azeotropic distillation with toluene and a water collector. Solvent removal under reduced pressure afforded the title compound as a low melting slightly yellow solid. Crystals appropriate for data collection were obtained by slow diffusion of diethyl ether into a chloroform solution under an inert atmosphere.

### Refinement

The carbon bound H atoms were placed in calculated positions and subsequently treated as riding with C—H distances of 0.99 Å and *U*_iso_(H) = 1.2*U*eq(C). The hydrogen atoms located on N1 and N2 were located on a difference map and freely refined with individual isotropic temperature factors. The deepest hole in electron density (-0.33 e A^-3^) is located at a distance of 0.94 Å from C5.

### Crystal data

**Table:**

C_14_H_32_N_6_	*Z* = 1
*M**_r_* = 284.46	*F*(000) = 158
Triclinic, *P*1	*D*_x_ = 1.18 Mg m^−^^3^
Hall symbol: -P 1	Mo *K*α radiation, λ = 0.71073 Å
*a* = 6.2732 (3) Å	Cell parameters from 6758 reflections
*b* = 6.4988 (3) Å	θ = 1.0–27.5°
*c* = 10.7152 (6) Å	µ = 0.08 mm^−^^1^
α = 99.751 (2)°	*T* = 150 K
β = 93.115 (2)°	Block, colourless
γ = 110.410 (3)°	0.4 × 0.28 × 0.28 mm
*V* = 400.45 (3) Å^3^	

### Data collection

**Table:**

Bruker–Nonius KappaCCD diffractometer	1806 independent reflections
Radiation source: fine-focus sealed tube	1599 reflections with *I* > 2σ(*I*)
graphite	*R*_int_ = 0.099
φ and ω scans	θ_max_ = 27.5°, θ_min_ = 1.9°
Absorption correction: multi-scan (*SORTAV*; Blessing, 1995)	*h* = −7→8
*T*_min_ = 0.649, *T*_max_ = 0.985	*k* = −8→8
4952 measured reflections	*l* = −13→13

### Refinement

**Table:**

Refinement on *F*^2^	Primary atom site location: structure-invariant direct methods
Least-squares matrix: full	Secondary atom site location: difference Fourier map
*R*[*F*^2^ > 2σ(*F*^2^)] = 0.068	Hydrogen site location: inferred from neighbouring sites
*wR*(*F*^2^) = 0.208	H atoms treated by a mixture of independent and constrained refinement
*S* = 1.23	*w* = 1/[σ^2^(*F*_o_^2^) + (0.0859*P*)^2^ + 0.2591*P*] where *P* = (*F*_o_^2^ + 2*F*_c_^2^)/3
1806 reflections	(Δ/σ)_max_ < 0.001
99 parameters	Δρ_max_ = 0.30 e Å^−^^3^
0 restraints	Δρ_min_ = −0.33 e Å^−^^3^

### Special details

**Table:**

Geometry. All esds (except the esd in the dihedral angle between two l.s. planes) are estimated using the full covariance matrix. The cell esds are taken into account individually in the estimation of esds in distances, angles and torsion angles; correlations between esds in cell parameters are only used when they are defined by crystal symmetry. An approximate (isotropic) treatment of cell esds is used for estimating esds involving l.s. planes.
Refinement. Refinement of *F*^2^ against ALL reflections. The weighted *R*-factor wR and goodness of fit *S* are based on *F*^2^, conventional *R*-factors *R* are based on *F*, with *F* set to zero for negative *F*^2^. The threshold expression of *F*^2^ > σ(*F*^2^) is used only for calculating *R*-factors(gt) etc. and is not relevant to the choice of reflections for refinement. *R*-factors based on *F*^2^ are statistically about twice as large as those based on *F*, and *R*- factors based on ALL data will be even larger.

### Fractional atomic coordinates and isotropic or equivalent isotropic displacement parameters (Å^2^)

**Table:**

	*x*	*y*	*z*	*U*_iso_*/*U*_eq_	
N1	0.1344 (3)	0.5390 (3)	0.32247 (17)	0.0229 (4)	
H1	0.145 (5)	0.533 (5)	0.233 (3)	0.033 (7)*	
N2	0.6399 (3)	0.5507 (3)	0.29452 (16)	0.0202 (4)	
H2	0.775 (5)	0.583 (4)	0.312 (2)	0.019 (6)*	
N3	0.1962 (3)	0.1707 (3)	0.15019 (15)	0.0200 (4)	
C1	0.3489 (3)	0.6779 (3)	0.4061 (2)	0.0231 (5)	
H1A	0.3671	0.5978	0.4744	0.028*	
H1B	0.3328	0.819	0.4475	0.028*	
C2	0.5687 (3)	0.7378 (3)	0.3420 (2)	0.0234 (5)	
H2A	0.5468	0.808	0.2696	0.028*	
H2B	0.6945	0.8513	0.404	0.028*	
C3	0.5883 (3)	0.4564 (3)	0.15762 (18)	0.0234 (5)	
H3A	0.7345	0.473	0.1221	0.028*	
H3B	0.5167	0.5461	0.1168	0.028*	
C4	0.4308 (3)	0.2106 (3)	0.12080 (18)	0.0225 (5)	
H4A	0.4267	0.1575	0.0282	0.027*	
H4B	0.4954	0.1211	0.1664	0.027*	
C5	0.1748 (3)	0.1640 (3)	0.28616 (18)	0.0211 (5)	
H5A	0.329	0.2224	0.3355	0.025*	
H5B	0.0966	0.0072	0.2955	0.025*	
C6	0.0377 (3)	0.3056 (3)	0.33779 (19)	0.0230 (5)	
H6A	−0.1207	0.2366	0.2934	0.028*	
H6B	0.0293	0.3037	0.4296	0.028*	
C7	0.0265 (4)	−0.0249 (3)	0.06487 (18)	0.0241 (5)	
H7A	−0.1166	−0.0741	0.1049	0.029*	
H7B	0.0849	−0.1493	0.0528	0.029*	

### Atomic displacement parameters (Å^2^)

**Table:**

	*U*^11^	*U*^22^	*U*^33^	*U*^12^	*U*^13^	*U*^23^
N1	0.0170 (8)	0.0243 (9)	0.0240 (9)	0.0054 (7)	0.0015 (6)	0.0006 (7)
N2	0.0140 (8)	0.0237 (9)	0.0189 (8)	0.0049 (6)	0.0007 (6)	−0.0013 (6)
N3	0.0168 (8)	0.0211 (8)	0.0152 (8)	0.0016 (6)	0.0000 (6)	−0.0021 (6)
C1	0.0182 (9)	0.0242 (10)	0.0229 (10)	0.0068 (8)	0.0025 (7)	−0.0037 (8)
C2	0.0190 (9)	0.0193 (9)	0.0276 (10)	0.0033 (7)	0.0038 (7)	0.0009 (8)
C3	0.0199 (9)	0.0266 (10)	0.0183 (9)	0.0033 (8)	0.0038 (7)	0.0010 (8)
C4	0.0205 (10)	0.0246 (10)	0.0181 (9)	0.0061 (8)	0.0024 (7)	−0.0028 (7)
C5	0.0209 (9)	0.0214 (9)	0.0166 (9)	0.0034 (7)	0.0007 (7)	0.0018 (7)
C6	0.0174 (9)	0.0243 (10)	0.0226 (10)	0.0028 (7)	0.0043 (7)	0.0016 (8)
C7	0.0231 (10)	0.0193 (9)	0.0205 (10)	−0.0007 (7)	−0.0025 (7)	−0.0014 (8)

### Geometric parameters (Å, °)

**Table:**

N1—C6	1.466 (3)	C3—C4	1.526 (3)
N1—C1	1.475 (2)	C3—H3A	0.99
N1—H1	0.96 (3)	C3—H3B	0.99
N2—C2	1.460 (3)	C4—H4A	0.99
N2—C3	1.462 (2)	C4—H4B	0.99
N2—H2	0.80 (3)	C5—C6	1.524 (3)
N3—C7	1.464 (2)	C5—H5A	0.99
N3—C4	1.464 (2)	C5—H5B	0.99
N3—C5	1.477 (2)	C6—H6A	0.99
C1—C2	1.531 (3)	C6—H6B	0.99
C1—H1A	0.99	C7—C7^i^	1.525 (4)
C1—H1B	0.99	C7—H7A	0.99
C2—H2A	0.99	C7—H7B	0.99
C2—H2B	0.99		
			
C6—N1—C1	114.96 (17)	H3A—C3—H3B	107.5
C6—N1—H1	106.1 (17)	N3—C4—C3	113.41 (16)
C1—N1—H1	114.6 (17)	N3—C4—H4A	108.9
C2—N2—C3	117.19 (17)	C3—C4—H4A	108.9
C2—N2—H2	111.5 (17)	N3—C4—H4B	108.9
C3—N2—H2	106.5 (18)	C3—C4—H4B	108.9
C7—N3—C4	112.85 (15)	H4A—C4—H4B	107.7
C7—N3—C5	112.41 (15)	N3—C5—C6	109.75 (16)
C4—N3—C5	112.25 (15)	N3—C5—H5A	109.7
N1—C1—C2	116.37 (17)	C6—C5—H5A	109.7
N1—C1—H1A	108.2	N3—C5—H5B	109.7
C2—C1—H1A	108.2	C6—C5—H5B	109.7
N1—C1—H1B	108.2	H5A—C5—H5B	108.2
C2—C1—H1B	108.2	N1—C6—C5	113.78 (16)
H1A—C1—H1B	107.3	N1—C6—H6A	108.8
N2—C2—C1	115.49 (16)	C5—C6—H6A	108.8
N2—C2—H2A	108.4	N1—C6—H6B	108.8
C1—C2—H2A	108.4	C5—C6—H6B	108.8
N2—C2—H2B	108.4	H6A—C6—H6B	107.7
C1—C2—H2B	108.4	N3—C7—C7^i^	112.1 (2)
H2A—C2—H2B	107.5	N3—C7—H7A	109.2
N2—C3—C4	115.55 (17)	C7^i^—C7—H7A	109.2
N2—C3—H3A	108.4	N3—C7—H7B	109.2
C4—C3—H3A	108.4	C7^i^—C7—H7B	109.2
N2—C3—H3B	108.4	H7A—C7—H7B	107.9
C4—C3—H3B	108.4		
			
C6—N1—C1—C2	106.9 (2)	C7—N3—C5—C6	−97.75 (19)
C3—N2—C2—C1	101.1 (2)	C4—N3—C5—C6	133.75 (17)
N1—C1—C2—N2	−67.9 (2)	C1—N1—C6—C5	−71.3 (2)
C2—N2—C3—C4	−118.6 (2)	N3—C5—C6—N1	−56.6 (2)
C7—N3—C4—C3	151.78 (17)	C4—N3—C7—C7^i^	−77.7 (3)
C5—N3—C4—C3	−79.9 (2)	C5—N3—C7—C7^i^	154.1 (2)
N2—C3—C4—N3	67.4 (2)		

Symmetry codes: (i) −*x*, −*y*, −*z*.

### Hydrogen-bond geometry (Å, °)

**Table:**

*D*—H···*A*	*D*—H	H···*A*	*D*···*A*	*D*—H···*A*
N2—H2···N1^ii^	0.80 (3)	2.37 (3)	3.129 (3)	159 (2)

Symmetry codes: (ii) *x*+1, *y*, *z*.
